# Premenstrual Dysphoric Disorder: A Cross-Sectional Study on Prevalence and Awareness Among Medical Students

**DOI:** 10.7759/cureus.80917

**Published:** 2025-03-20

**Authors:** Razan R Babour, Sarah A Alharbi, Sarah M Alzahrani, Amjad A Alshareef, Lina S Bazeeb, Rania Zahid, Nadia El Amin, Nouf K Alalshaikh, Rami Ahmad

**Affiliations:** 1 Department of Internal Medicine, College of Medicine, King Saud bin Abdulaziz University for Health Sciences, King Abdullah International Medical Research Center, Jeddah, SAU; 2 Department of Basic Medical Sciences, College of Medicine, King Saud bin Abdulaziz University for Health Sciences, King Abdullah International Medical Research Center, Jeddah, SAU; 3 Psychiatry Section, Department of Medicine, College of Medicine, Ministry of National Guard - Health Affairs, Jeddah, SAU; 4 Department of Western Region, King Abdullah International Medical Research Center, Jeddah, SAU

**Keywords:** health education and awareness, premenstrual dysphoric disorder (pmdd), premenstrual syndrome (pms), psychiatry and mental health, women's mental health

## Abstract

Background: Premenstrual dysphoric disorder (PMDD), a severe form of PMS, has been recognized recently as a mental disorder. PMDD can present with disabling physical, mental, and psychological symptoms affecting women's daily functions. Due to its overlapping nature with other disorders, the prevalence of PMDD remains unknown. This study aimed to evaluate the awareness of PMDD among medical students and to assess the prevalence of PMDD among female students.

Methods: This cross-sectional study included 377 medical students from King Saud bin Abdulaziz University for Health Sciences, Jeddah. PMDD awareness was assessed using a self-developed questionnaire, and prevalence among women was evaluated with the PSST. Data were analyzed using the John's Macintosh Project software, version 10.0 (JMP Statistical Discovery, LLC, Cary, NC), with chi-square, Mann-Whitney, and Kruskal’s tests, considering p < 0.05 as statistically significant.

Results: Out of 377 students, there was a significant difference in recognizing PMDD as a mental disorder between 106 female students (66%) and 51 male students (27.3%). Significantly higher PMDD awareness was observed among students in the clinical years and those with medical or mental conditions. Around 40% of the students had a positive attitude toward PMDD, believing it may require medical management. Among the 150 female medical students who met the inclusion criteria for PSST assessment to screen for PMDD, 8.7% tested positive, and this was correlated with the severity of dysmenorrhea. Anxiety/tension was the most common symptom in the PMDD group, with significant impairment in social activities and home responsibilities.

Conclusion: The study demonstrated a noticeable level of awareness toward PMDD among medical students, especially among women and those in the clinical years. The association between PMDD symptoms and the severity of dysmenorrhea highlights the relationship between hormonal fluctuations and PMDD manifestations. Early detection and intervention are required to improve the quality of life for women with PMDD. Further research is recommended to assess the awareness of practitioners and the general population about the diagnosis of PMDD and to explore PMDD pathophysiology.

## Introduction

Premenstrual dysphoric disorder (PMDD) was included in the Diagnostic and Statistical Manual of Mental Disorders (DSM-5) and the International Classification of Diseases, 11th Revision (ICD-11) as a full diagnostic category 10 years ago [1,2]. The World Health Organization defines PMDD in the ICD-11 as "a pattern of mood symptoms (depressed mood, irritability), somatic symptoms (lethargy, joint pain, overeating), or cognitive symptoms (concentration difficulties, forgetfulness) that begin several days before the onset of menses" [2].

Premenstrual syndrome (PMS) comprises a variety of mental and psychological symptoms experienced by approximately 90% of women during the luteal phase of their menstrual cycle. Despite the prevalence of PMS, less than 10% received a diagnosis of PMDD [3]. PMDD represents a severe form of PMS, characterized by severe symptoms that greatly impair daily living [3]. These symptoms can arise at any time between menarche and menopause [4,5]. PMDD can generally occur in the last week before menstruation, improve within a few days after the start of menstruation, and be mild or absent in the subsequent weeks, and the symptoms must have been present for the majority of the previous year’s menstrual cycles [4,5]. PMDD can have a great impact, leading to both physical and mental impairment, in addition to social and occupational dysfunction [4]. Women diagnosed with PMS are more likely to experience increased work absenteeism and impairment in work, school, and household responsibilities [3]. Moreover, PMDD can lead to lower self-esteem, a sense of inadequacy, dissatisfaction, and a more sedentary lifestyle [4]. Moreover, women who experience PMDD have a higher risk of suicide than the general population [6].

The etiology of PMDD is still unidentified, but the research in this area is highly active [7]. Some studies pointed out that the cause of PMDD is due to abnormalities in neurotransmitters, mainly changes in the levels of gonadal steroid hormones, which can play an important role in developing some PMDD symptoms [7]. Several studies have claimed that the symptoms are caused by cyclical fluctuations in estrogen and progesterone levels [7]. Likewise, it has been proposed that nutritional factors and the deficiency of vitamins can be associated with it [3]. Nonetheless, further studies are needed to clarify the exact causes of the development of PMDD [3].

Although the pathophysiology of PMDD is not definitively established yet, different treatment strategies for PMDD depend on the suggested theories, and they aim mainly to control the symptoms. A concise review by Carlini and Deligiannidis indicates the significant effects of selective serotonin reuptake inhibitors (SSRIs), an antidepressant, as a first-line treatment. Oral contraceptives (OCPs) are the second-line treatments as they were shown to effectively reduce PMDD symptoms. In case of poor response to SSRIs and OCPs, leuprolide, a gonadotropin-releasing hormone agonist that induces ovarian suppression, can also be used as a last resort due to its severe side effects [8].

A study conducted in Brazil (2018) showed that 17.6% of 727 young women experience PMDD [9]. In Ethiopia, a study with a population of 254 women found a high prevalence of PMDD (66.9%) [10]. Moreover, the study found that the degree of dysmenorrhea was statistically associated with PMDD. A recent study done in India showed that the prevalence of PMDD among 661 medical and paramedical students was 5.04% [4]. Additionally, it presented fatigue/lack of energy and physical symptoms (e.g., breast tenderness, headache, and bloating) as the two most common PMDD symptoms, respectively. Another study found that the prevalence of PMDD among 254 Jordanian women aged between 18 and 45 was 10.2% [11]. According to a Saudi study conducted at Umm al-Qura University, the prevalence was 36.6% among female medical students [12].

Moreover, as it is newly classified as a distinct diagnostic disorder, a lack of awareness regarding the nature of PMDD is expected. According to a 2018 study conducted in Karachi among 448 female university students, the majority (96.4%) were aware of PMS; however, only 19% were aware of PMDD [13]. Hence, it is essential to increase the awareness of PMDD among the general population and medical students to reduce the number of undiagnosed cases and enhance the quality of life for women affected by this condition. Therefore, this study aimed to evaluate the awareness of PMDD among female and male medical students. Additionally, it aimed to assess the prevalence of PMDD and to investigate its associated factors among female medical students.

## Materials and methods

Study design, area, and settings

A cross-sectional quantitative study was performed at the College of Medicine (COM-J), King Saud bin Abdulaziz University for Health Sciences (KSAU-HS), Jeddah, Saudi Arabia. The study targeted medical students (male and female students) during the academic year 2021-2022. All students in phases 2 and 3 were encouraged to participate in the study (phase 2 involved preclinical medical students in their third and fourth years, while phase 3 involved clinical medical students in their fifth and sixth years). Students in phase 1 (first and second years) were excluded as they were in the preparatory years before joining the medical college. The study was conducted online, with validated self-administered questionnaires distributed to students via their university emails. A convenience sampling technique was used.

Two different calculations for the sample size were applied to serve the study objectives. To evaluate PMDD awareness, the sample size required was 262, which was calculated using the Raosoft Online Sample Size Calculator (Raosoft, Inc., Seattle, WA) with a confidence interval of 95%, a margin of error of 5%, and a population size of 813 (total number of male and female students at COM-J). The sample size required to estimate the prevalence of PMDD symptoms was 84, calculated using the Epitools Sample Calculator (Ausvet, Australia) with a confidence level of 95%, a 5% margin of error, and considering that there were 318 female medical students and an estimated PMDD prevalence of 5.8%, according to DSM-5 [1].

Since the premenstrual symptoms screening tool (PSST) follows DSM-5 criteria, more exclusion criteria were used in the study to fulfill DSM-5 criteria for estimating the prevalence of PMDD symptoms. After collecting all the female responses to the survey, exclusion was made to all participants who had a history of any chronic medical conditions (like thyroid disorders, polycystic ovarian syndrome) or a history of any mental condition (major depressive disorder, panic disorder, persistent depressive disorder, or a personality disorder) or were under a current treatment that could influence PMDD.

Ethical consideration

A written consent form was attached at the beginning of the questionnaires. To ensure the participants’ anonymity, personal information such as names, phone numbers, or student ID numbers was not asked in the questionnaire. The Institutional Review Board at King Abdullah International Medical Research Center in Jeddah approved the study with approval number H-01-R-005 (study no: SP21J/028/02).

Development of the questionnaire

The questionnaire used in this study was in English and had two versions: one for male students and the other for female students (see the Appendix). The targeted medical students were expected to possess sufficient English capability to comprehend the questionnaire. The male version included two sections: demographics and awareness level assessment, whereas the female version contained two additional sections about reproductive characteristics and premenstrual screening. The questions were multiple-choice and dichotomous. The questionnaire was meticulously designed following extensive literature research.

*Demographic Q* *uestions*

The first section of the questionnaire in both versions collected demographic information from the participants, including age, academic year, residency, marital status, parenthood, and a history of mental illness. In the female version, additional questions were added, including weight, height, smoking, alcohol consumption, contraceptive use, number of previous pregnancies, current medications, history of mental illness, and history of medical conditions.

Awareness Level of PMDD

The second section of the questionnaire in both versions aimed to assess the awareness of PMDD among medical students. This section contained nine questions. The first six questions covered general knowledge regarding aspects that are affected during or before the menstrual cycles, including mood changes, appetite, quality and quantity of sleep, experiencing social withdrawal, and decreased productivity in daily activities, like work, school, and hobbies. A question was added to assess participants' awareness and understanding of PMDD as a medical condition. A further question was added to ascertain the knowledge of PMDD as a recognized disorder according to DSM-5. The last question was about the possibility of seeking medical management if the symptoms were noticed in a friend or a relative (or female participants). Participants were provided with options to answer "yes," "no," or "I don’t know." A correct answer was assigned with a score of one, reflecting that the participants acquired the necessary knowledge regarding a particular aspect of the question. On the other hand, an incorrect answer received a score of zero, suggesting a lack of knowledge or a lack of understanding. Eventually, a cumulative score will be calculated out of 9 for each participant to compare discrepancies between both genders. Moreover, correct answers to a question were considered as a positive attitude, whereas a false answer to a question was considered as a negative attitude. After conducting a pilot study, Cronbach's alpha for reliability was calculated for this section and was 0.513, suggesting a good level of reliability [14].

Reproductive Characteristics

The third section of the female version of the questionnaire assessed reproductive characteristics such as age at first cycle, average menstrual cycle length, menstrual cycle regularity, number of bleeding days per cycle, amount of bleeding, presence of dysmenorrhea, and severity of dysmenorrhea.

Premenstrual Symptoms Screening

The fourth section was included only in the females' version of the questionnaire, where two items regarding a previous diagnosis of PMDD and the presence of a family history of PMDD were added. Moreover, the PSST, a validated instrument used to assess the prevalence of the symptoms of PMDD, was also included [14]. According to Steiner et al., the PSST reflects and translates categorical DSM-4 criteria into a rating scale with degrees of severity [9]. Furthermore, PSST is considered a fast and effective starting point for measuring the severity and impact of premenstrual symptoms through which female participants might meet the criteria of PMDD.

The PSST consists of 14 items assessing various premenstrual symptoms and five items rating functional impairment due to symptoms. Participants must rate each item according to the intensity and presence of symptoms, with one of four options given: not at all, mild, moderate, or severe.

A positive screening score for PMDD is indicated by meeting the following criteria: at least one of the following four items must be rated as severe (irritability, anxiety, tearfulness/increased sensitivity to rejection, and depressed mood), at least four out of the 14 questions must be reported as moderate or severe, and at least one severe functional impairment. All of these criteria must be present for the diagnosis of PMDD. For the diagnosis of moderate to severe PMS, the following criteria must be present: at least one of the first four items (irritability, anxiety, increased sensitivity to rejection, and depressed mood) is moderate to severe, at least four of the 14 items are moderate to severe, and at least one moderate to severe functional impairment.

Data analysis

Data analysis was performed using the John's Macintosh Project software, version 10.0 (JMP Statistical Discovery, LLC, Cary, NC). Percentages and frequencies were used to describe categorical variables, such as gender, marital status, and reproductive characteristics of female participants. The median and the interquartile range (IQR) were used to present nonnormally distributed variables. The chi-square test was used to assess the relationship of PMDD with multiple factors. The Mann-Whitney test was used to evaluate the difference in awareness between women and men. The Kruskal test assessed the association between awareness and the PSST score. A p value less than 0.05 was considered statistically significant.

## Results

Sociodemographic characteristics of respondents

Out of 377 students, 190 female and 187 male students agreed to participate in this study, resulting in an overall response rate of 46.37%. The demographic characteristics of the participants are summarized in Table 1. The median age of both male and female participants was 21 years. For female participants, the IQR was 20-23 years, while for male participants, the IQR was 20-22 years. Thirty-four female students reported using some medications, while 156 (82%) did not use any. The most commonly used medications were antidepressants and antianxiety medications, with 34 out of 190 female participants (17.89%) reporting their use. A history of mental illnesses was reported by 28 female (14%) and eight male (4%)participants. The main reported mental illnesses among female participants were generalized anxiety disorder 15 (7.8%), depression 11 (5.7%), and social anxiety disorder 4 (2%). Regarding medical history, 22 female participants (11%) reported having chronic medical conditions, with polycystic ovarian syndrome and hypothyroidism being the most common conditions. Among the male participants, 155 individuals (82%) reported having sisters, while 32 individuals (17%) indicated that they did not have any sisters.

**Table 1:** Sociodemographics for participants BMI: body mass index

Variables	Females (n = 190), n (%)	Males (n = 187), n (%)
Academic year		
Preclinical year	114 (60)	142 (75)
Clinical year	76 (40)	45 (24)
Residency		
Lived alone	6 (3)	-
Family	184 (96)	-
Marital status		
Single	164 (86)	173 (92)
In a relationship or engaged	20 (10)	13 (6)
Married	5 (2)	1 (0.5)
Divorced	1 (0.52)	-
Having children	2 (1)	2 (1)
Having sisters	-	155 (82)
BMI		
Underweight: <18.5 kg/m^2^	35 (18)	-
Normal: 18.5-24.99 kg/m^2^	112 (58)	-
Overweight: ≥25 kg/m^2^	43 (22)	-
Smoker	12 (6)	
Using a contraceptive method	5 (2)	-
Using medications	34 (17)	-
History of mental illness	28 (14)	8 (4)
History of medical condition	22 (11)	-

PMDD awareness among the participants

**Figure 1:**
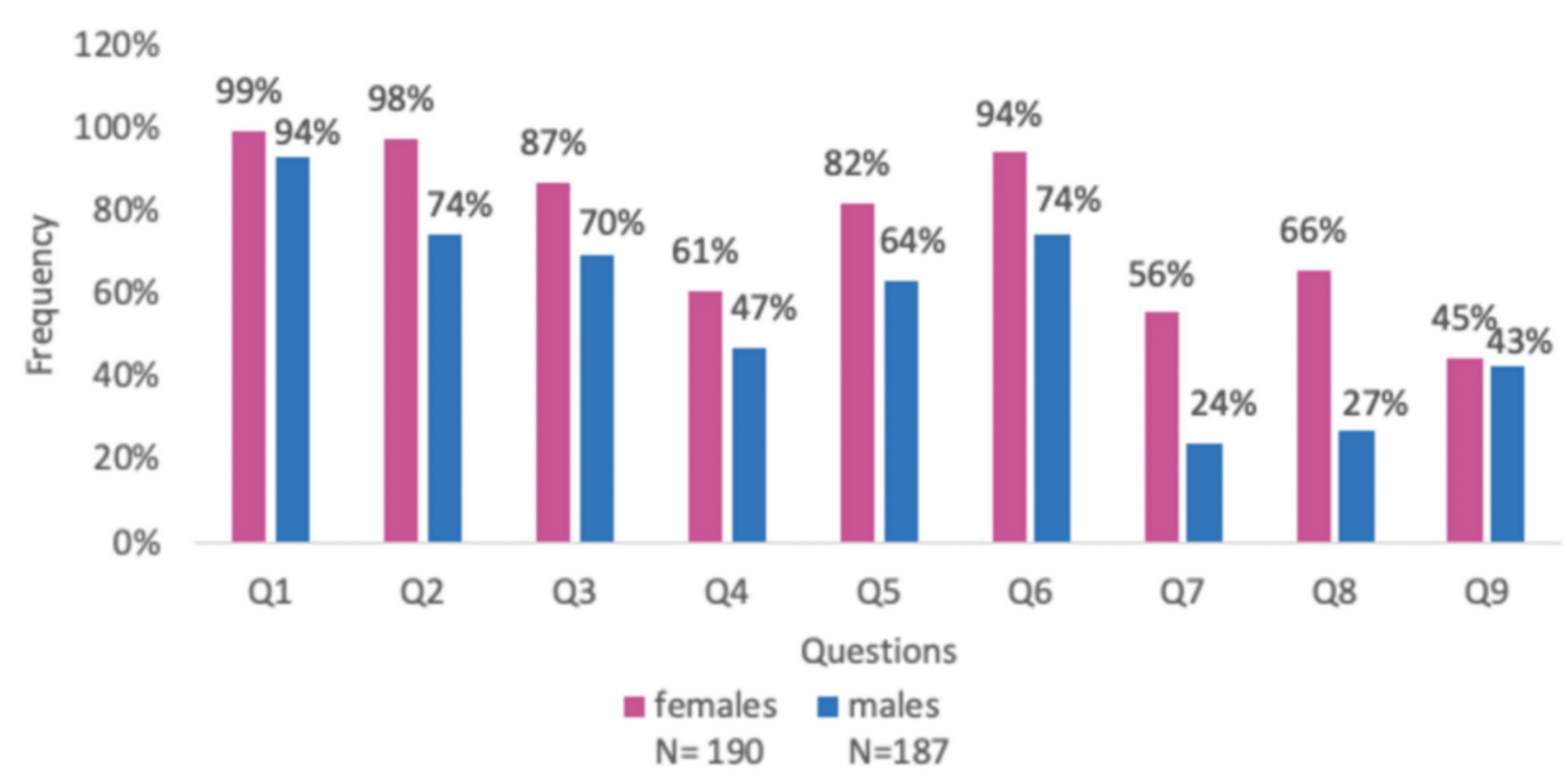
Frequencies of participants who obtained the correct answer for each question regarding the awareness about PMDD Q1: Could women experience mood changes before or during their menstrual cycle? Q2: Could women experience loss or an increase in their appetite before or during their menstrual cycle? Q3: Can the quality and quantity of sleep be affected before or during the menstrual cycle? Q4: Do women not experience social withdrawal before or during their menstrual cycle? Q5: Is daily productivity affected by the menstrual cycle? Q6: Could women suffer from severe emotional and physical symptoms up to the point that it affects their daily functioning before or during their menstrual cycle? Q7: Is the group of emotional and physical symptoms that affect women’s daily functioning during or before their menstrual cycles considered a disorder? Q8: Is there a diagnostic name for these emotional and physical disturbances during or before the menstrual cycle, specifically premenstrual dysphoric disorder? Q9: If you encountered these symptoms or noticed them in a close friend/relative, would you consider or recommend seeking medical management? PMDD: premenstrual dysphoric disorder

The PMDD awareness questions and the frequency of correct answers are shown in Figure 1. Since the awareness score is not normally distributed, the Mann-Whitney test has been used to compare the awareness of male and female participants, displaying higher awareness among female participants. Most participants correctly answered the first question about experiencing mood changes before or within the menstrual cycle. However, the question that was the least answered correctly by female participants (44.7%) was question 9, about considering seeking medical management, and by male participants (24.1%) was question 7, considering the emotional and physical symptoms during or before their menstrual cycles as a disorder. There was a significant difference in response between female and male participants to question 8, which assessed the knowledge of PMDD as a disorder according to DSM-5. One hundred six female participants (66%) answered positively, compared to 51 male participants (27.3%). Regarding question 9, reflecting the attitude toward PMDD symptoms, 85 female (44.7%) and 80 male (42.7%) participants demonstrated a positive attitude, as illustrated in Figure 1.

Furthermore, the Mann-Whitney test showed a significant difference in the awareness score between academic years (p < 0.001). Hence, awareness is high among clinical students compared to preclinical students. Additionally, statistically significant differences were observed in participants with a history of mental illnesses (p < 0.001). Moreover, the history of medical conditions was correlated with a significant difference in score (p = 0.0196), thus indicating that female students with a history of mental and/or medical conditions had higher awareness. The Kruskal test indicated that participants with PMDD had significantly higher awareness (p < 0.001). No associations were found with other variables.

Reproductive characteristics of female respondents

The median age at the onset of menstruation (menarche) was 12.5 years. Regarding the menstrual cycle length, 54 students (28%) reported having cycles of 25 days or less, while 136 (71%) had cycles lasting more than 25 days. Moreover, 34 (17%) had irregular menstrual cycles, in contrast to 156 (82%) participants who had regular cycles. Regarding the duration of bleeding, 133 students (70%) reported having bleeding for six days or less, whereas 57 (30%) had bleeding for more than six days. Regarding the amount of bleeding, only three students (1%) had spots, 96 (50%) had mild, 78 (41%) had moderate, and 13 (6%) had severe bleeding. Dysmenorrhea was reported in 69 students (36%). Of those, the severity of dysmenorrhea was mild in 40 students (21%), moderate in 69 students (36%), and severe in 33 students (17%). Four students reported a history of PMS, while 186 (97%) reported no PMS. A history of PMDD was reported by only three students. A positive family history of PMDD was present in 10 female students (5%), while 180 (94%) had no family history of PMDD.

Prevalence and associated factors of PMDD

Out of 190 female participants, 150 were screened using the PSST tool, while 40 were excluded due to an underlying medical or mental history or current treatment that could influence PMDD. Of the 150 included female participants, thirteen (8.66%) were diagnosed with PMDD, 66 (44%) experienced moderate to severe PMS, and 71 (47%) had no/mild PMS.

As presented in Table 2, anxiety/tension was the most common symptom in the PMDD group, reported by all 13 female participants (100%). In the moderate/severe PMS group, fatigue/lack of energy was the most common, noted by 53 females (80.30%). In the no/mild PMS group, fatigue/lack of energy was the most common symptom, stated by 30 female participants (42.25%). Across all groups, insomnia was the least common symptom. Significant differences in symptom prevalence were noticed among the three groups.

**Table 2:** Frequency of premenstrual symptoms among the three groups according to PSST PMS: premenstrual syndrome; PMDD: premenstrual dysphoric disorder; PSST: premenstrual symptoms screening tool

Symptoms	No/mild PMS (n = 71), n (%)	Moderate/severe PMS (n = 66), n (%)	PMDD (n = 13), n (%)	Chi-square	p value
Anger/irritability	28 (39.44)	52 (78.79)	12 (92.31)	28.09	<0.0001
Anxiety/tension	17 (23.94)	47 (71.21)	13 (100)	44.08	<0.0001
Tearfulness	24 (33.80)	42 (63.64)	11 (84.62)	18.50	<0.0001
Depressed mood	20 (28.17)	47 (71.21)	12 (92.31)	34.39	<0.0001
Decreased interest in work	18 (25.35)	45 (68.18)	8 (61.54)	26.32	<0.0001
Decreased interest in home activities	15 (21.13)	46 (69.70)	7 (53.85)	32.98	<0.0001
Decreased interest in social activities	14 (19.72)	46 (69.70)	10 (76.92)	39.56	<0.0001
Difficulty concentrating	10 (14.08)	32 (48.48)	10 (76.92)	29.09	<0.0001
Fatigue/lack of energy	30 (42.25)	53 (80.30)	12 (92.31)	26.47	<0.0001
Overeating/food craving	29 (40.85)	42 (63.64)	7 (53.85)	7.14	0.02
Insomnia	5 (7.04)	9 (13.64)	4 (30.77)	6.16	0.0460
Hypersomnia	19 (26.76)	44 (66.67)	9 (69.23)	24.39	<0.0001
Feeling overwhelmed	15 (21.13)	47 (71.21)	11 (84.62)	41.71	<0.0001
Physical symptoms	23 (32.39)	48 (72.73)	12 (92.31)	30.38	<0.0001

In Table 3, statistically significant impairment was observed across all aspects in the PMDD group. The most common impairments among participants with PMDD were in social life activities and home responsibilities, reported by 12 individuals (92.31%). The second most common impairments occurred in family relationships and school efficiency, each noted by 11 participants (84.62%). Among the moderate/severe PMS group, the reported functional impairments were in school efficiency by 48 participants (72.73%), social life activities by 37 participants (56.06%), home responsibility by 36 participants (54.55%), relationship with family by 35 participants (53.03%), and relationship with classmates by 22 participants (33.33%).

**Table 3:** Frequency of functional impairment among the three groups according to PSST PMS: premenstrual syndrome; PMDD: premenstrual dysphoric disorder; PSST: premenstrual symptoms screening tool

Functional impairment item	No/mild PMS (n = 71), n (%)	Moderate/severe PMS (n = 66), n (%)	PMDD (n = 13), n (%)	Chi-square	p value
School efficiency	2 (2.82)	48 (72.73)	11 (84.62)	80.68	<0.0001
Relationship with classmates	1 (1.41)	22 (33.33)	8 (61.54)	35.76	<0.0001
Relationship with family	2 (2.82)	35 (53.03)	11 (84.62)	57.74	<0.0001
Social life activities	4 (5.63)	37 (56.06)	12 (92.31)	58.29	<0.0001
Home responsibility	3 (4.23)	36 (54.55)	12 (92.31)	60.16	<0.0001

No association between residency, marital status, BMI, or smoking and PMDD was found for the social demographic. For the reproductive characteristics, PMDD was associated with dysmenorrhea and its severity, as shown in Table 4. However, PMDD was not associated with the age of first menarche, average length or regularity of menstrual cycle, days of bleeding per cycle, or the amount of bleeding.

**Table 4:** Association with reproductive characteristics in the three groups according to PSST PMS: premenstrual syndrome; PMDD: premenstrual dysphoric disorder; PSST: premenstrual symptoms screening tool

Reproductive characteristics	n (%)	No PMS, n (%)	Severe PMS, n (%)	PMDD, n (%)	p value
Age of first menarche					
≤12.5	73 (48.67)	32 (45.07)	32 (48.48)	9 (69.23)	0.27
>12.5	77 (51.33)	39 (54.93)	34 (51.52)	4 (30.77)	
The average length of the menstrual cycle					
≤25 days	48 (32)	17 (23.94)	24 (36.36)	7 (53.85)	0.06
>25 days	102 (68)	54 (76.06)	42 (63.64)	6 (46.15)	
Regularity of menstrual cycle					
Regular	124 (82.67)	60 (84.51)	53 (80.3)	11 (84.62)	0.79
Irregular	26 (17.33)	11 (15.49)	13 (19.7)	2 (15.38)	
Approximate days of bleeding per one cycle					
≤6 days	107 (71.33)	51 (71.83)	47 (71.21)	9 (69.23)	0.98
>6 days	43 (28.67)	20 (28.17)	19 (28.79)	4 (30.77)	
Amount of bleeding					
Spotting	3 (2)	2 (2.82)	1 (1.52)	0 (0)	0.21
Mild	76 (50.67)	41 (57.75)	32 (48.48)	3 (23.08)	
Moderate	62 (41.33)	26 (36.62)	27 (40.91)	9 (69.23)	
Heavy	9 (6)	2 (2.82)	6 (9.09)	1 (7.69)	
Dysmenorrhea	94 (62.67)	36 (50.7)	47 (71.21)	11 (84.62)	0.012
Severity of dysmenorrhea					
Mild	24 (16)	17 (23.94)	7 (10.61)	0 (0)	<0.0001
Moderate	45 (30)	12 (16.9)	30 (45.45)	3 (23.08)	
Severe	25 (16.67)	7 (9.86)	10 (15.15)	8 (61.54)	

## Discussion

PMDD is a severe form of PMS recently recognized in the DSM-5 as a distinct depressive disorder with its diagnostic criteria [1]. This study demonstrated higher awareness among female medical students. Notably, there was a lack of existing literature on PMDD awareness, except for one study assessing PMS awareness among female medical students in Pakistan, where only 19% had previous knowledge about PMDD [13]. Additionally, this study revealed that students in the clinical phase, regardless of gender, exhibited higher awareness, likely due to the studied curriculum. Furthermore, female participants with a history of mental or medical conditions, as well as those who screened positive for PMDD, were more aware of PMDD. However, over half of the participants did not consider recommending or seeking medical management when noticing PMDD symptoms in female participants themselves or in their close friends/relatives. This can indicate a negative attitude toward PMDD symptoms, possibly due to the stigma around mental illnesses or underestimating the severity of the symptoms. The current study showed that the prevalence of PMDD symptoms among 150 participants was 8.67%, moderate to severe PMS was 44%, and no to mild PMS was 47%. These results are congruent with the worldwide estimated prevalence of PMDD, ranging from 3% to 9% [15-19]. A Jordanian study recorded the prevalence of PMDD as 10.2%, which is slightly higher than our results [11]. Various studies have found higher prevalence, ranging from 20.4% to 36.6%, compared to our study [12,20,21]. The difference between the present study and other studies can be attributed to the sample selection since our study targeted only medical students with an average age of 21 and used different assessment tools.

Our findings noted that the participants in the PMDD group reported anxiety/tension as the most common symptom, followed by mood symptoms (anger/irritability, depressed mood, feeling overwhelmed, fatigue/lack of energy), accompanied by physical symptoms as the second most common symptom. In line with these findings, many cross-sectional studies have revealed significant results regarding the frequency of these symptoms [4,12,20,21]. A case-control study reported a strong association between an elevated level of psychological stress and neuroticism with PMDD and PMS [22]. The PMS group recorded fatigue/lack of energy, anger/irritability, and physical symptoms in the current study. Comparable results were also reported in the studies by Hamaideh et al. and Chumpalova et al. [11,23]. Compared to the PMS group, the PMDD group showed noteworthy results regarding the frequency of all symptoms. The least reported symptom by all groups during the luteal and menstrual phases was insomnia, which followed the findings of Thakrar et al., Goweda et al., and Daşıkan [4,12,20]. However, a study conducted by Khazaie et al. showed a significant report regarding insomnia in women with PMDD [24]. Lin et al. found a significant association with insomnia in women with PMDD that was worsened in the late luteal phase [25]. One possible cause is the fluctuation of sex hormones (progesterone, estrogen), leading to a disturbance in the circadian rhythm and decreased response to melatonin, leading to insomnia [26,27]. However, the difference between our study and the mentioned studies can be related to the special tools used to assess sleep quality and PMDD.

In the current study, the most common functional impairments in the PMDD group were found to be in social life activities and home responsibility, followed by relationships with family and school efficiency. Meanwhile, in the PMS group, the most frequent functional impairments were in school efficiency and social life activities. Nevertheless, the PMDD group showed a marked statistical functional impairment in all areas. Our results are comparable to those from Thakrar et al. and Daşıkan studies [4,20].

Our study did not find an association between PMDD and residency, marital status, BMI, smoking, and usage of any contraceptive method since there are no great differences between the categories. Some of these factors can be explained by the cultural background of female participants, as it is not common for single women in our community to live alone or to use contraceptive methods. In addition, the participants were medical students, and most of them were still single. However, a previous case-control study revealed that women who smoke are at a higher risk of developing PMS or PMDD [28]. Regarding the BMI, most of the female participants were within the normal range of BMI; hence, no association was found between BMI and PMDD. Another study suggested an association between higher BMI and PMDD [29]. Moreover, our results indicated that there is no association between PMDD and reproductive characteristics, including the age of menarche, the average length of the menstrual cycle, regularity of the menstrual cycle, amount of bleeding, and approximate days of bleeding per one cycle. Regardless, the study found a significant association between the severity of dysmenorrhea and PMDD, which is congruent with other studies [10,21].

One of the limitations of this study is the use of a self-reported questionnaire, which might be subject to self-bias that resulted in overestimation or underestimation of PMS or PMDD symptoms among the participants. Another limitation of the current study is the use of a retrospective measurement, while the prospective measurement can be more accurate in diagnosing PMDD. The study was conducted among medical students as a targeted population with a small sample size and a low response rate; thus, it cannot be generalized to the general population. Although our study found a high association between some factors that manifested in the development of PMDD symptoms, causation cannot be established and requires further research with a larger sample size and additional investigation. Despite the limitation, the results of this study are nonetheless valid for answering our research question.

This study offers valuable insights and demonstrates several strengths and implications in the research area of PMDD. It highlights the gap in research regarding PMDD awareness among medical students, emphasizing the notable difference in knowledge between male and female students. Additionally, the higher awareness among clinical-phase students can be attributed to their curriculum, providing a strong connection between academic exposure and recognition of the disorder. The study’s reliability is reinforced by the use of validated screening tools, and it draws a clear distinction between PMDD and PMS, shedding light on the severity of symptoms and the associated functional impairments. A significant finding is the correlation between PMDD and dysmenorrhea, suggesting that women experiencing severe menstrual pain should be thoroughly assessed for PMDD. The research further illustrates that PMDD can lead to considerable disruptions in social, academic, and domestic roles, emphasizing its profound impact on quality of life. With a prevalence rate of 8.67%, the study’s findings align with global estimates, emphasizing the disorder’s significance among young women. The results also highlight the importance of medical education in raising awareness, particularly among male students, to enhance their understanding and support for women with PMDD. Although awareness is relatively high, many participants showed reluctance to seek medical intervention, pointing to the potential stigma surrounding PMDD or an underestimation of its severity. Further research should aim for larger, longitudinal studies to explore the causes of PMDD and its relationship to other mood disorders, along with examining its neurobiological basis. The study also calls for broader PMDD awareness among the general public and healthcare providers.

## Conclusions

In conclusion, the current study found that the prevalence of PMDD symptoms was 8.67% among female medical students. Furthermore, there was a statistically significant association between PMDD and dysmenorrhea. Moreover, the study demonstrated a high awareness among female medical students compared to male students at KSAU-HS. Future studies should consider investigating the impact of reproductive characteristics on PMDD and the level of awareness among both practitioners and the general population on the diagnosis and management of PMDD. Furthermore, prospective research is needed to assess the prevalence of PMDD. An array of avenues is necessary to understand PMDD's etiology and its similarities and differences with PMS and other mood disorders.

## Appendices

A questionnaire to measure prevalence and awareness of PMDD among medical students at King Saud bin Abdulaziz University for Health Sciences on Jeddah branch, Saudi Arabia (cross-sectional study)

Version A (Females Only)

**Table 5:** Social demographics

No.	Question	Options
1.	Age	☐ 19-21 ☐ 22-24 ☐ 25 and more
2.	Academic year	☐ 3rd year ☐ 4th year ☐ 5th year ☐ 6th year
3.	Residency	☐ Alone ☐ With family
4.	Marital status	☐ Single ☐ Engaged ☐ Married ☐ Divorced ☐ Widowed
5.	Do you have children?	☐ No ☐ Yes
6.	Number of previous pregnancies	-
7.	Weight	-
8.	Height	☐ No ☐ Yes
9.	Are you a smoker?	☐ No ☐ Yes
10.	Do you consume alcohol?	☐ No ☐ Yes
11.	Are you using any contraceptive method?	☐ No ☐ Yes
12.	Are you currently on any medication?	☐ No ☐ Yes
13.	If yes, please mention your medications	-
14.	Do you have any history of mental illnesses?	☐ No ☐ Yes
15.	If yes, please mention your diagnosis	-
16.	Do you have any history of medical conditions?	☐ No ☐ Yes
17.	If yes, please mention it	-

**Table 6:** Awareness

No.	Question	Yes	No	I don’t know
1.	Do you think that women could experience mood changes before or during their menstrual cycle?	-	-	-
2.	Do you think that women could experience loss or increase in their appetite before or during their menstrual cycle?	-	-	-
3.	Do you think that the quality and quantity of sleep can be affected before or during the menstrual cycle?	-	-	-
4.	Do you think that women do not experience social withdrawal before or during their menstrual cycle?	-	-	-
5.	Do you think that daily productivity is not affected by the menstrual cycle?	-	-	-
6.	Do you think that women could suffer from severe emotional and physical symptoms up to the point that it affects their daily function before or within their menstrual cycle?	-	-	-
7.	Do you think that the group of emotional and physical symptoms that affect women's daily function during or before their menstrual cycles is considered a disorder?	-	-	-
8.	Do you think that there is a diagnostic name, which is premenstrual dysphoric disorder, for these emotional and physical disturbances during or before the menstrual cycle?	-	-	-
9.	Do you think that if you encountered these symptoms or noticed them in a close friend/relative, would you consider or recommend seeking medical management?	-	-	-

**Table 7:** Reproductive characteristics

No.	Question	Options
1.	Age at first menarche in years	☐ 12 or before ☐ 13-14 ☐ 15-16 ☐ 17 and more
2.	Average length of the menstrual cycle	☐ 20 or less ☐ 21-25 ☐ 26-30 ☐ 31-35 ☐ More than 35
3.	Regularity of the menstrual cycle	☐ Regular ☐ Irregular
4.	Approximate days of bleeding per cycle	☐ 1-3 ☐ 4-6 ☐ 7-9 ☐ More than 10
5.	Amount of bleeding	☐ Spotting ☐ Mild ☐ Moderate ☐ Heavy
6.	Do you have dysmenorrhea?	☐ No ☐ Yes
7.	Severity of dysmenorrhea, if you have it	☐ I answered “no” ☐ Mild ☐ Moderate ☐Severe

**Table 8:** Prevalence PMDD: premenstrual dysphoric disorder

No.	Question	Options
1.	Have you ever been diagnosed with PMDD?	☐ No ☐ Yes
2.	Do you have a family history of PMDD?	☐ No ☐ Yes

**Table 9:** The premenstrual screening tool The following criteria must be present for a diagnosis of PMDD: 1) at least one of #1, #2, #3, or #4 is severe, 2) in addition, at least four of #1 to #14 are moderate to severe, and 3) at least one of A, B, C, D, or E is severe The following criteria must be present for a diagnosis of moderate to severe PMS: 1) at least one of #1, #2, #3, or #4 is moderate to severe, 2) in addition, at least four of #1 to #14 are moderate to severe, and 3) at least one of A, B, C, D, or E is moderate to severe PMS: premenstrual syndrome; PMDD: premenstrual dysphoric disorder

Symptom	Not at all	Mild	Moderate	Severe
Do you experience some or any of the following premenstrual symptoms that start before your period and stop within a few days of bleeding?				
Anger/irritability	-	-	-	-
Anxiety/tension	-	-	-	-
Tearful/increased sensitivity to rejection	-	-	-	-
Depressed mood/hopelessness	-	-	-	-
Decreased interest in work activities	-	-	-	-
Decreased interest in home activities	-	-	-	-
Decreased interest in social activities	-	-	-	-
Difficulty concentrating	-	-	-	-
Fatigue/lack of energy	-	-	-	-
Overeating/food cravings	-	-	-	-
Insomnia	-	-	-	-
Hypersomnia (needing more sleep)	-	-	-	-
Feeling overwhelmed or out of control	-	-	-	-
Physical symptoms: breast tenderness, headaches, joint/muscle pain, bloating, weight gain	-	-	-	-
Have your symptoms, as listed above, interfered with?				
Your work efficiency or productivity	-	-	-	-
Your relationships with coworkers	-	-	-	-
Your relationships with your family	-	-	-	-
Your social life activities	-	-	-	-
Your home responsibilities	-	-	-	-

Version M (Males Only)

**Table 10:** Social demographics

No.	Question	Options
1.	Age	☐ 19-21 ☐ 22-24 ☐ 25 and more
2.	Academic year	☐ 3rd year ☐ 4th year ☐ 5th year ☐ 6th year
3.	Marital status	☐ Single ☐ Engaged ☐ Married ☐ Divorced ☐Widowed
4.	Do you have children?	☐ No ☐ Yes
5.	Do you have any history of mental illnesses?	☐ No ☐ Yes
6.	If yes, please mention your diagnosis	-

**Table 11:** Awareness

No.	Question	No	I don't know	Yes
1.	Do you experience mood changes before or during your menstrual cycle?	-	-	-
2.	Do you experience a loss or increase in their appetite before or during your menstrual cycle?	-	-	-
3.	Can the quality and quantity of sleep be affected before or within their menstrual cycle?	-	-	-
4.	Do not experience social withdrawal before or within their menstrual cycle?	-	-	-
5.	Is daily productivity not affected by the menstrual cycle?	-	-	-
6.	Do you suffer from severe emotional and physical symptoms up to the point that it affects their daily functions before or within the menstrual cycle?	-	-	-
7.	Did you know that the group of emotions and physical symptoms that affect women daily function during or before their menstrual cycles have been classified officially as a disorder under the name of premenstrual dysphoric disorder?	-	-	-
8.	If you encountered the above symptoms or noticed them in a close friend/relative, would you consider or recommend seeking medical management?	-	-	-
